# Two novel species of *Alternaria* (*Pleosporales*, *Pleosporaceae*) from cowpea (*Fabaceae*) in China

**DOI:** 10.3897/mycokeys.138.205866

**Published:** 2026-08-18

**Authors:** Shen-Xian Zhou, Jia-Hui Huang, Shi-Qiang Chen, Qian Deng, Cai-Hua Shi, Quan-Zhou Wen, Pian-Pian Yan, Jian-Xin Deng

**Affiliations:** 1 Department of Plant Protection, College of Agriculture, Yangtze University, Jingzhou 434025, China Department of Plant Protection, College of Agriculture, Yangtze University Jingzhou China; 2 Institute of Advanced Agricultural Science, Hubei University of Arts and Science, Xiangyang 441054, China Institute of Advanced Agricultural Science, Hubei University of Arts and Science Xiangyang China; 3 Agriculture and Rural Affairs Bureau of Lichuan City, Lichuan 445400, China Agriculture and Rural Affairs Bureau of Lichuan City Lichuan China

**Keywords:** *
Alternaria
*, cowpea, morphology, novel species, phylogeny

## Abstract

The genus *Alternaria* (*Pleosporales*, *Pleosporaceae*) is globally distributed, occurs on a wide variety of substrates, and comprises saprobic, endophytic, and pathogenic species. In this study, four unidentified *Alternaria* isolates were obtained from symptomatic leaves of cowpea (*Vigna unguiculata*) in Hainan Province, China. These *Alternaria* spp. were characterized based on morphological features and phylogenetic analysis with multiple loci (*GAPDH*, *RPB2*, *Alt a 1*, *EndoPG*, and OPA10-2). Based on the combined morphological and phylogenetic evidence, two novel species in *Alternaria* sect. *Alternaria* are described: *A. unguiculatae***sp. nov**. and *A. hainanensis***sp. nov**. This study expands the known diversity of *Alternaria* associated with *Fabaceae*, particularly cowpea, in China.

## Introduction

*Alternaria* is a taxonomically complex genus of dematiaceous fungi, currently comprising more than 790 species epithets and over 360 accepted species distributed among 30 sections (Li et al. 2023; Ahmadpour et al. 2025; Yang et al. 2025). Members of the genus occupy diverse ecological niches as endophytes, saprobes, and pathogens on a wide range of substrates and hosts, including many economically important crops (Thomma 2003). Traditionally, *Alternaria* species were morphologically divided into large-spored and small-spored taxa (Simmons 2007). In Simmons’ morphology-based system, approximately 128 small-spored morphospecies were assigned to 10 subsections (Simmons 2007; Aung et al. 2024). However, small-spored taxa are among the most taxonomically challenging members of the genus because closely related taxa frequently overlap in conidial shape, septation, beak morphology, and conidial catenation (Woudenberg et al. 2013). This problem is particularly evident in *Alternaria* sect. *Alternaria*, where multilocus and genomic analyses supported only 11 phylogenetic species and one species complex, while 35 morphospecies delimited on morphological grounds were synonymized under *A. alternata* (Woudenberg et al. 2015). Together, these studies highlight the limited discriminatory power of morphology alone in delimiting small-spored *Alternaria* taxa (Lawrence et al. 2013; Woudenberg et al. 2015).

DNA-based taxonomy of *Alternaria* has relied on multilocus datasets derived from both ribosomal and protein-coding loci (Jayawardena et al. 2019a, 2019b; Nwe et al. 2024; Chen et al. 2026; He et al. 2026). However, single-locus datasets often lack sufficient resolution to delineate species boundaries across all sections of the genus, particularly in small-spored groups such as *Alternaria* sect. *Alternaria* and *Alternaria* sect. *Infectoriae* (Peever et al. 2004; Andrew et al. 2009). Although certain loci may be informative in specific contexts, their phylogenetic utility is not uniform across all taxonomic groups in *Alternaria*. For instance, *Alt a 1* is useful for species identification, whereas the plasma membrane ATPase and CAL loci can improve molecular delimitation in some groups (Hong et al. 2005; Lawrence et al. 2013). Consequently, multilocus approaches have become essential for resolving species boundaries and higher-level relationships in *Alternaria* (Dettman and Eggertson 2021; Bhushan et al. 2025; Yang et al. 2025). At a broader taxonomic scale, Woudenberg et al. (2015) used SSU, LSU, ITS, *GAPDH*, *RPB2*, and *TEF1* to redefine phylogenetic lineages within *Alternaria* and allied genera and to establish a modern sectional framework for the genus (Woudenberg et al. 2015; Gannibal 2019; Gannibal et al. 2022). Currently, whole-genome comparisons have further improved the delimitation of ambiguous taxa, especially in morphologically conserved groups (Lawrence et al. 2016).

Cowpea (*Vigna unguiculata* (L.) Walp.) is an important leguminous crop widely cultivated in tropical and subtropical regions, with a global harvested area exceeding 15 million hectares and an annual dry grain production of approximately 9 million metric tons (Horn and Shimelis 2020; Abebe and Alemayehu 2022; Kim et al. 2025). Several *Alternaria* taxa have been reported from cowpea and related cultivated forms of *Vigna unguiculata*, including *A. vignae* from China (Huang et al. 2022), *A. cassiae* from South Africa (La Grange and Aveling 1998), *A. malorum* from Iran (Atashi Khalilabad and Fotouhifar 2022), and *A. alternata* from yardlong bean in India (Roy et al. 2024). During the investigation of fungal diseases on cowpea leaves in Hainan Province, China, two small-spored *Alternaria* taxa were recovered that could not be assigned to any known species. Their taxonomic status was evaluated using morphology, five-locus phylogeny (*GAPDH*, *RPB2*, *Alt a 1*, *EndoPG*, and OPA10-2), species-delimitation methods, and phylogenetic network analyses. This study aims to establish their species boundaries and strengthen the taxonomy of cowpea-associated small-spored *Alternaria*. However, the currently known records of *Alternaria* on cowpea remain limited, and the species-level taxonomy of the associated isolates is still poorly resolved (Li et al. 2023; Schmey et al. 2023).

## Materials and methods

### Fungal isolation

Diseased leaves of cowpea (*Vigna unguiculata*) exhibiting leaf spot symptoms were collected from Haikou, Hainan Province, China (20°01'07"N, 110°20'56"E), in 2024. Small tissue segments (approximately 1 cm × 1 cm) containing both diseased and healthy tissues were excised from the margins of typical single-symptom lesions, divided into several equal pieces, sequentially surface sterilized in 75% ethanol for 1 min and 2% sodium hypochlorite for 30 s, rinsed three times with sterile distilled water, and then placed on potato dextrose agar (PDA, BD Difco, Montreal, Canada). Plates were incubated at 25 °C for 5–7 days, after which single-spore isolates were obtained. A total of 105 pure fungal cultures were obtained and provisionally assigned to 20 genera through preliminary morphological and molecular identification, including 36 cultures of *Alternaria*. Four focal cultures, representing two putative species, were selected for further taxonomic assessment because they could not be confidently assigned to any described species during preliminary screening. The focal cultures were YzU 241394, YzU 241395, YzU 241428, and YzU 241429. Each pair comprised duplicate cultures derived from the same original isolate. Pure cultures were maintained on PDA, potato carrot agar (PCA), and V8 juice agar (V8A) for subsequent morphological observations and were preserved on PDA slants at 4 °C and in glycerol tubes at –80 °C. Representative cultures were deposited in the Fungi Herbarium of Yangtze University, Jingzhou, China.

### Morphology

Colony diameter, texture, surface and reverse pigmentation, and the presence of diffusible pigments were recorded. Micromorphological features were examined under a Nikon ECLIPSE Ni-U light microscope (Nikon, Japan) at magnifications of ×400–1000. Photomicrographs were taken from structures mounted in lactophenol picric acid solution (LPA). For each isolate, at least 30 conidia and conidiophores were randomly selected and measured. Single-spore isolations were obtained on water agar (WA) by transferring individual germinating conidia to PDA for purification. Pure cultures were subsequently maintained on PDA, PCA, and V8A. Cultural characteristics were assessed on PDA, PCA, and V8A after incubation at 25 °C for 7 days.

### DNA extraction, PCR amplification, and sequencing

Fresh mycelia were obtained from fungal strains cultured on PDA and processed for genomic DNA extraction following a previously described protocol (Watanabe et al. 2010). The extracted DNA was used as a template for amplification of five gene regions, including *GAPDH*, *RPB2*, *Alt a 1*, *EndoPG*, and OPA10-2 (Woudenberg et al. 2015). Polymerase chain reaction (PCR) amplification of the above-mentioned regions was performed using a Bio-Rad T100™ Thermal Cycler with the primer pairs gpd1/gpd2 (Berbee et al. 1999), RPB2-5F/RPB2-7c (Liu et al. 1999), Alt-for/Alt-rev (Hong et al. 2005), PG3/PG2b (Andrew et al. 2009), and OPA10-2L/OPA10-2R (Andrew et al. 2009), respectively. PCR reaction mixtures and thermal cycling conditions followed established protocols described in previous studies (Nwe et al. 2024). PCR products were purified and bidirectionally sequenced using Sanger sequencing by a commercial sequencing service. Sequence chromatograms were inspected and edited using sequence-editing software, and consensus sequences were assembled using SeqMan Pro v. 17.3 (DNASTAR, Inc., Madison, WI, USA). All consensus sequences were deposited in GenBank, with accession numbers provided in Table 1. The establishment of new species followed current recommendations for fungal species delimitation (Jeewon 2016; Hyde et al. 2023).

**Table 1.** GenBank accession numbers of *Alternaria* spp. used for phylogenetic analysis.

**Species**	**Strain**	***Alt a 1***	***GAPDH***	***RPB2***	**OPA10**–**2**	***EndoPG***
*A. alstroemeriae*	CBS 118808	KP123845	KP124153	KP124764	KP124601	KP123993
*A. alstroemeriae*	CBS 118809^T^	MH084526	KP124154	KP124765	KP124602	KP123994
*A. alternantherae*	CBS 124392	KP123846	KC584096	KC584374	–	–
*A. alternata*	CBS 916.96^T^	AY563301	AY278808	KC584375	–	–
*A. alternata*	CBS 112249	KP123886	KP124192	KP124806	KP124648	KP124039
*A. arborescens*	CBS 119544^T^	KP123955	JQ646321	KP124878	KP124722	KP124112
*A. arborescens*	CBS 102605^T^	AY563303	AY278810	KC584377	KP124712	AY295028
*A. arctoseptata*	MFLUCC 21-0139^T^	OK236755	OK236702	OK236655	–	–
*A. baoshanensis*	MFLUCC 21-0124^T^	OK236760	OK236706	OK236659	–	–
*A. betae-kenyensis*	CBS 118810^T^	KP123966	KP124270	KP124888	KP124733	KP124123
*A. breviconidiophora*	MFLUCC 21-0786^T^	OK236751	OK236698	OK236651	–	–
*A. burnsii*	CBS 118816	KP123970	KP124273	KP124892	KP124737	KP124127
*A. burnsii*	CBS 118817	KP123971	KP124274	KP124893	KP124738	KP124128
*A. burnsii*	CBS 107.38^T^	KP123967	JQ646305	KP124889	KP124734	KP124124
*A. burnsii*	CBS 879.95	KP123969	KP124272	KP124891	KP124736	KP124126
*A. burnsii*	CBS 130264	KP123972	KP124275	KP124894	KP124739	KP124129
*A. burnsii*	CBS 110.50	KP123968	KP124271	KP124890	KP124735	KP124125
*A. burnsii*	CBS 108.27	KP123850	KC584162	KC584468	KP124605	KP123997
*A. eichhorniae*	CBS 489.92^T^	KP123973	KP124276	KP124895	KP124740	KP124130
*A. ellipsoidialis*	MFLUCC 21-0132	OK236743	OK236690	OK236643	–	–
*A. eupatoriicola*	MFLUCC 21-0122	OK236736	OK236683	OK236636	–	–
*A. gaisen*	CBS 118488^R^	KP123975	KP124278	KP124897	KP124743	KP124132
*A. gaisen*	CBS 632.93^R^	KP123974	KC584116	KC584399	KP124742	AY295033
*A. gossypina*	CBS 104.32^T^	JQ646395	JQ646312	KP124900	KP124746	KP124135
*A. gossypina*	CBS 102601	KP123979	KP124282	KP124903	KP124749	KP124138
*A. iridiaustralis*	CBS 118487	KP123982	KP124285	KP124906	KP124752	KP124141
*A. iridiaustralis*	CBS 118486^T^	KP123981	KP124284	KP124905	KP124751	KP124140
*A. jacinthicola*	CBS 878.95	KP123983	KP124286	KP124907	KP124753	KP124142
*A. jacinthicola*	CPC 25267	KP123985	KP124288	KP124909	KP124755	KP124144
*A. jacinthicola*	CBS 133751^T^	KP123984	KP124287	KP124908	KP124754	KP124143
*A. jingzhouensis*	YZU 221144^T^	OR887694	OR887690	OR887688	OR887684	OR887692
*A. koreana*	SPL 2-1^T^	LC631831	LC621647	LC621681	LC631857	LC631844
*A. lathyri*	MFLUCC 21-0140^T^	OK236728	OK236675	OK236628	–	–
*A. ljiangensis*	YZU 221458^T^	OQ686781	OQ686785	OQ686789	OQ686787	OQ686779
*A. longipes*	CBS 540.94^R^	AY563304	AY278811	KC584409	KP124758	KP124147
*A. longipes*	CBS 121332^R^	KP123989	KP124292	KP124913	KP124760	KP124149
*A. longxiensis*	YZU 221221^T^	OQ473629	OQ512732	OQ543009	OQ543003	OQ512720
*A. lycopersici*	YZU 221185^T^	OQ473633	OQ512736	OQ543013	OQ543007	OQ512724
*A. macilenta*	MFLUCC 21-0138^T^	OK236726	OK236673	OK236626	–	–
*A. macroconidia*	MFLUCC 21-0134^T^	OK236757	OK236704	OK236657	–	–
*A. minimispora*	MFLUCC 21-0127^T^	OK236734	OK236681	OK236634	–	–
*A. momordicae*	YZU 161378	OR887695	OR887691	OR887689	OR887685	OR887693
*A. muriformispora*	MFLUCC 21-0784^T^	OK236730	OK236677	OK236630	–	–
*A. myanmarensis*	YZU 231736^T^	OR979657	OR963612	PP508256	PP034184	OR979663
*A. oblongoellipsoidea*	MFLUCC 22-0074^T^	OK236721	OK236668	OK236621	–	–
*A. obpyriconidia*	MFLUCC 21-0121^T^	OK236732	OK236680	OK236633	–	–
*A. orobanches*	MFLUCC 21-0137^T^	OK236763	OK236710	–	–	–
*A. oryzicola*	YZU 231199^T^	PV155522	PV155536	PV155548	PV155542	–
*A. ovoidea*	MFLUCC 21-0782^T^	–	OK236708	OK236661	–	–
*A. phragmiticola*	MFLUCC 21-0125^T^	OK236749	OK236696	OK236649	–	–
*A. poae*	YZU 231197^T^	PV155524	PV155538	PV155550	PV155544	PV155532
*A. poae*	YZU 231198	PV155523	PV155537	PV155549	PV155543	PV155531
*A. rostroconidia*	MFLUCC 21-0136^T^	OK236723	OK236670	OK236623	–	–
*A. salicicola*	MFLUCC 22-0072^T^	OK236753	OK236700	OK236653	–	–
*A. solanicola*	YZU 221189^T^	OQ473631	OQ512734	OQ543011	OQ543005	OQ512722
*A. tomato*	CBS 103.30	KP123991	KP124294	KP124915	KP124762	KP124151
*A. tomato*	CBS 114.35	KP123992	KP124295	KP124916	KP124763	KP124152
*A. torilis*	MFLUCC 14-0433^T^	OK236741	OK236688	OK236641	–	–
*A. yamethinensis*	YZU 231739^T^	OR979655	OR963610	PP179253	PP034182	OR979661
*A. zeae*	YZU 231602^T^	PV155521	PV155535	PV155547	PV155541	–
*A. zeae*	YZU 231638	PV155520	PV155534	PV155546	PV155540	–
*A. nelumbicola*	YZU 231505	PP793758	PP793752	PP793761	PP793767	PP793764
*A. nelumbicola*	YZU 231506	PP793759	PP793753	PP793762	PP793768	PP793765
***A. hainanensis* sp. nov**.	**YzU 241394^T^**	**PZ466122**	**PZ446769**	**PZ466126**	**PZ466130**	**PZ466132**
***A. hainanensis* sp. nov**.	**YzU 241395**	**PZ466123**	**PZ446770**	**PZ466127**	**PZ466131**	**PZ466133**
***A. unguiculatae* sp. nov**.	**YzU 241428**	**PZ446765**	**PZ446767**	**PZ466124**	**PZ466128**	**PZ446771**
***A. unguiculatae* sp. nov**.	**YzU 241429^T^**	**PZ446766**	**PZ446768**	**PZ466125**	**PZ466129**	**PZ446772**

Note: GenBank accession numbers of newly generated sequences are shown in bold. Ex-type strains are indicated by a superscript ^T^ and representative strains by a superscript R. Missing sequence data are indicated by an en dash (–).

### Phylogenetic analyses

Nucleotide sequences generated in this study were compared with those available in the NCBI nucleotide database using BLASTn (Altschul et al. 1990; Camacho et al. 2009). Reference sequences of *Alternaria* species included in the phylogenetic analyses were selected from recent publications (Table 1). Individual locus datasets were aligned using MAFFT v7.307 (online version) and subsequently trimmed with trimAl (Capella-Gutiérrez et al. 2009). Sequence assembly, alignment inspection, and concatenation of the five-locus dataset were carried out using MEGA 12.1.0 and the One-click Fungal Phylogenetic Tool (OFPT) v1.9.0 (Zeng et al. 2023; Stecher et al. 2026). The best-fit nucleotide substitution model for each locus was selected using ModelFinder under the Bayesian information criterion (BIC) (Kalyaanamoorthy et al. 2017). Within the OFPT workflow, the selected models were used for maximum likelihood (ML) analysis, while the corresponding MrBayes settings were generated for Bayesian inference (BI). ML, BI, and phylogenetic network analyses were conducted using IQ-TREE (Nguyen et al. 2015), MrBayes v3.2.7 (Ronquist et al. 2012), and SplitsTree 4.19.2 (Huson and Bryant 2006), respectively. ML branch support (BS) was assessed using 1,000 ultrafast bootstrap replicates (Hoang et al. 2018). Bayesian analyses were performed using a Markov chain Monte Carlo (MCMC) approach with four chains run for 50,000,000 generations and sampling every 100 generations. The first 25% of sampled trees were discarded as burn-in. Posterior probabilities (PP) were calculated from the remaining trees, and the BI consensus tree was generated when the average standard deviation of split frequencies decreased below 0.01.

## Results

### Phylogenetic analysis

A total of 67 strains representing species in *Alternaria* sect. *Alternaria*, including four cultures from this study representing two original isolates, were included in the phylogenetic analyses. Phylogenetic inference was based on five loci (*Alt a 1*, *EndoPG*, *GAPDH*, OPA10-2, and *RPB2*). Following alignment trimming, the individual datasets comprised 66 sequences and 472 characters for *Alt a 1*, 44 sequences and 447 characters for *EndoPG*, 67 sequences and 577 characters for *GAPDH*, 47 sequences and 627 characters for OPA10-2, and 66 sequences and 555 characters for *RPB2*. The best-fit nucleotide substitution models selected under the BIC were K2P+G4 for *Alt a 1*, TIMe+I for *EndoPG*, TN+F+G4 for *GAPDH*, and TNe+G4 for *OPA10-2* and *RPB2*.

**Figure 1.**
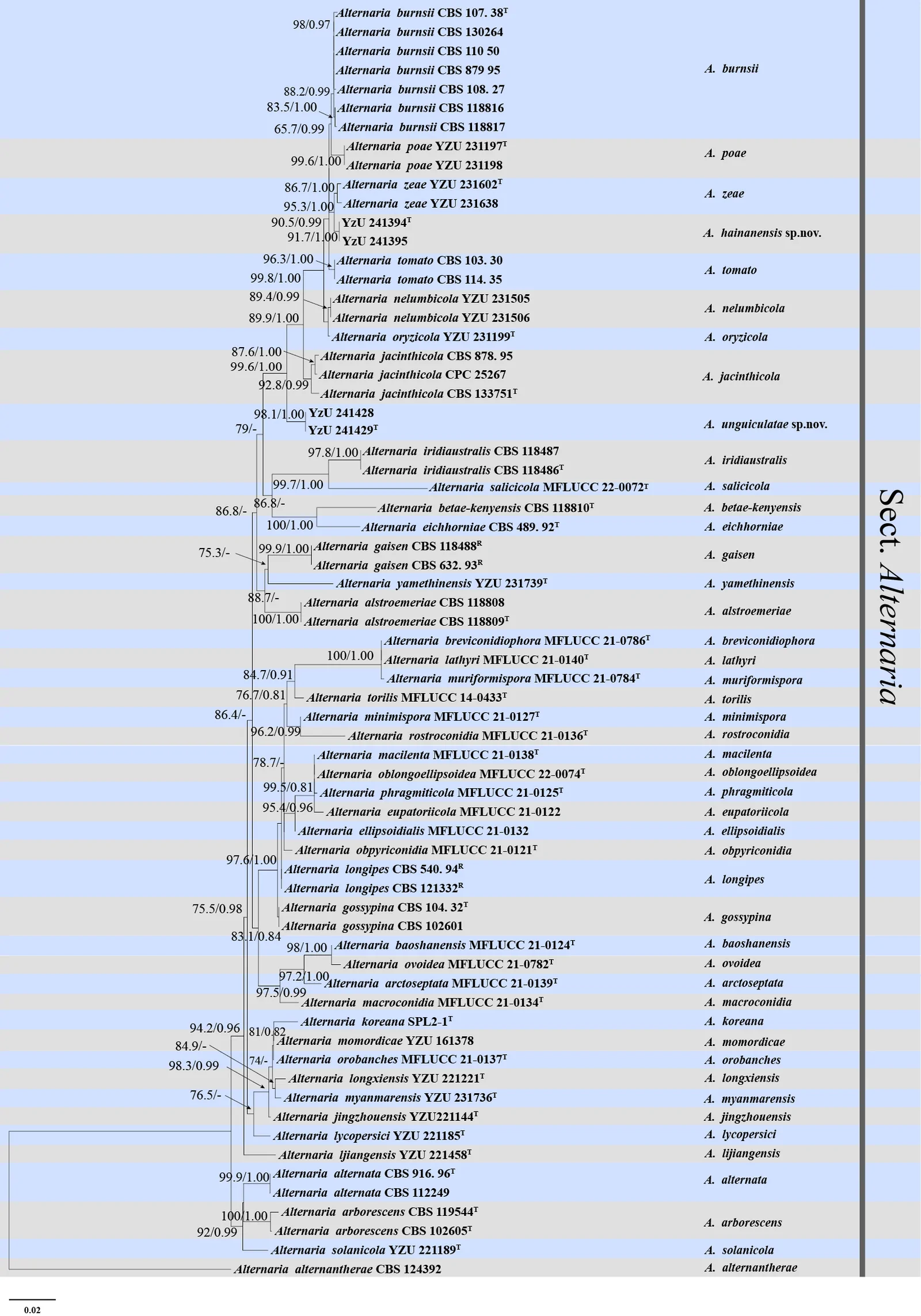
Phylogenetic tree constructed using the maximum likelihood method based on concatenated sequences of *GAPDH*, *RPB2*, *Alt a 1*, *EndoPG*, and OPA10-2 from *Alternaria* spp. Bootstrap support values (BS) and Bayesian posterior probabilities (PP) are given at the nodes (BS/PP). The strains from this study are marked in bold. Ex-type strains are indicated by a superscript T, and representative strains are indicated by a superscript R. *Alternaria alternantherae*CBS 124392 is used as the outgroup taxon.

The ML and BI analyses yielded largely congruent topologies. In the resulting phylogeny, the four strains examined in this study were all placed within *Alternaria* sect. *Alternaria* and clustered into two distinct, well-supported clades. Specifically, strains YzU 241428 and YzU 241429 formed a monophyletic clade with strong statistical support (BS/PP = 98.1/1.00), which was recovered as sister to *Alternaria jacinthicola* (CBS 878.95, CPC 25267, and CBS 133751^T^) (Fig. 1).

In addition, strains YzU 241394 and YzU 241395 grouped as a separate clade, supported by high nodal values (BS/PP = 95.3/1.00), and were resolved as closely related to *Alternaria zeae* (YZU 231602^T^ and YZU 231638) (Fig. 1). The clear phylogenetic separation of these two lineages from their closest relatives, together with their strong support values in the concatenated analyses, indicates that they represent two novel species within *Alternaria* sect. *Alternaria*.

### Taxonomy

#### Alternaria hainanensis

Taxon classificationFungiPleosporalesPleosporaceae

S.X. Zhou & J.X. Deng sp. nov.

AFDD36D8-8FA6-5EC0-B5CB-B72BFEEA385C

863711

Fig. 2

##### Etymology.

Named after Hainan, the locality from which the type specimen was collected.

**Figure 2.**
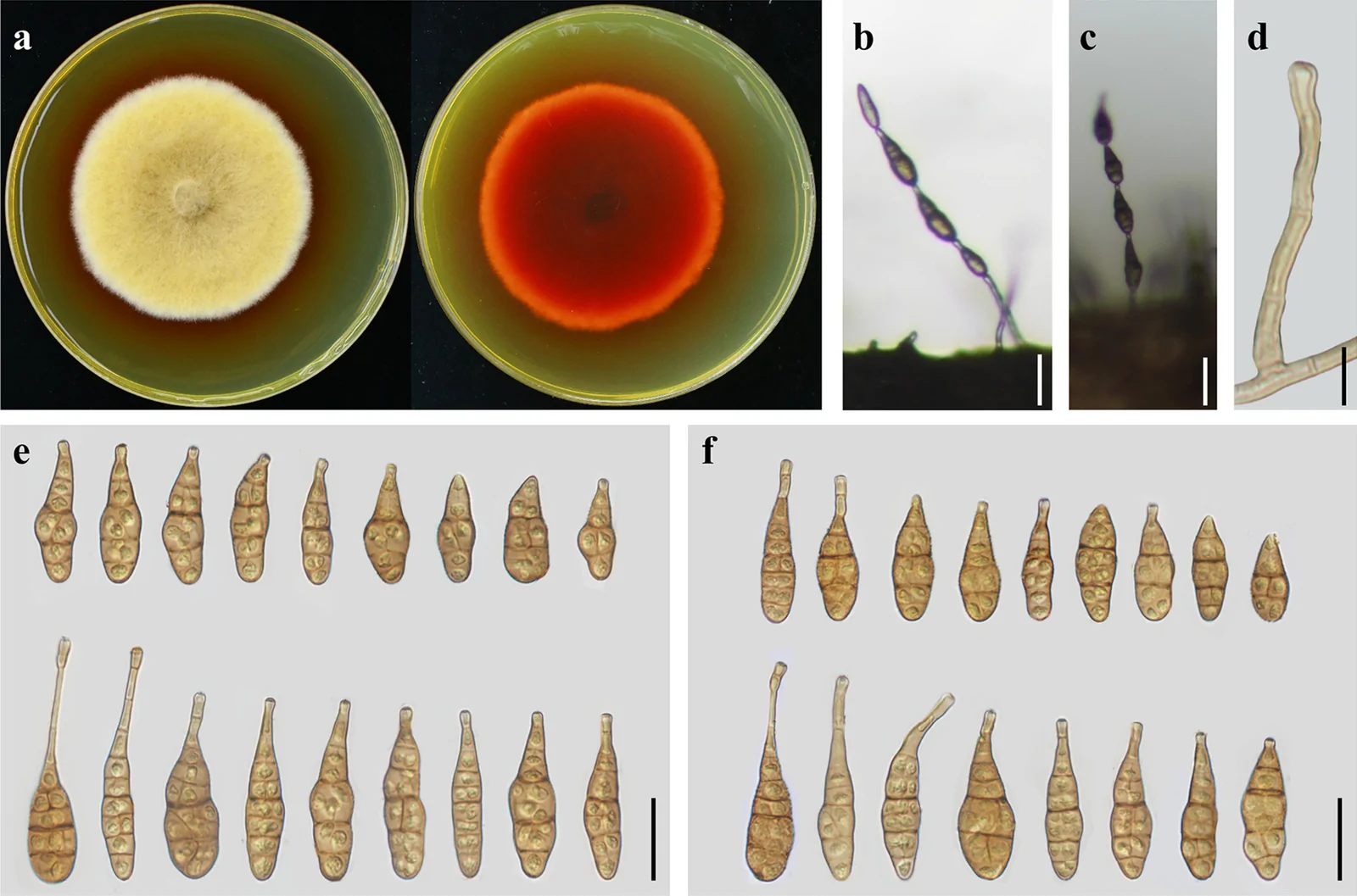
Morphology of *Alternaria hainanensis* sp. nov. (YzU 241394). **a**. Colony on PDA for 7 days at 25 °C; **b, c**. Sporulation on PCA; **d**. Sporulation on V8A; **e**. Conidia on PCA; **f**. Conidia on V8A. Scale bars: 50 µm (**b, c, d**); 25 µm (**e, f**).

##### Type.

China • Hainan Province, Haikou City, from diseased leaves of *Vigna unguiculata*; September 2024; S.X. Zhou; holotype YzU-H-2024-076B; ex-type culture YzU 241394; duplicate culture YzU 241395, derived from the same original isolate as the ex-type culture.

##### Description.

***Colonies*** on PDA (7 days at 25 °C in the dark) attaining 45–50 mm, circular, floccose to cottony, with abundant aerial mycelium, margin entire to slightly fimbriate, straw to buff, scarlet to red in reverse, have a greyish white margin. ***Conidiophores*** solitary or in small fascicles, arising from aerial hyphae, pale brown, smooth, septate, straight to slightly flexuous. ***Conidia*** formed in chains of 4–7, obclavate to long-obclavate, occasionally ellipsoid-obclavate, smooth-walled, pale brown to brown. On PCA, conidia 24–53 × 10–18 µm, with 3–6 transverse septa; beaks 7–33 × 3–5 µm, cylindrical to filiform. On V8A, conidia 32–55 × 12–20 µm, with 4–7 transverse septa; beaks 22–40 × 3–5 µm, elongate, often slightly swollen at the apex. Secondary septa occasionally present.

##### Notes.

Phylogenetically, strain YzU 241394 clustered in a well-supported clade closely related to *Alternaria zeae* (MLBS = 95.3%, BYPP = 1.00). Comparative sequence analyses revealed seven nucleotide differences across the sequenced gene regions between strain YzU 241394 and *A. zeae*, including 3 bp in *GAPDH* and 4 bp in *RPB2* (Suppl. material 1: table SS1). Morphologically, *A. zeae* produces conidia (26–46 × 10–18 µm) with apical beaks (9–93 × 2.5–4 µm) on PCA (Liu et al. 2025). In addition, *A. hainanensis* produces longer conidial chains, with 4–7 conidia per chain compared with 2–4 in *A. zeae* (Table 2). *A. hainanensis* is distinguished from *A. zeae* by longer conidia, longer swollen-tipped apical beaks, and orange-red pigment deposition on PDA, a trait absent in *A. zeae* colonies.

**Table 2.** Conidial morphology of *Alternaria* spp. from this study and their related species.

**Species**	**Conidia**				**Conidia per chain**	**Substrate**	**Reference**
	**Shape**	**Body size (µm)**	**Transverse septa**	**Beak size (µm)**			
*A. burnsii*	Ovoid to ellipsoid	30–50 × 9–13	5–8	–	solitary or short chain	Host	Simmons (2007)
	Narrow-ovoid to narrow-ellipsoid	30–40 × 8–14	3–7	–	–	PCA, V8A	
*A. poae*	Narrow-ovoid, subellipsoid, or obclavate	20–42 × 10–19	1–4	6–26 × 3–4	2–4	PCA	Liu et al. (2025)
		20–45 × 10–17	1–4	5–17 × 3–4	2–4	V8A	
*A. zeae*	Ovate, ellipsoid, or obclavate	26–46 × 10–18	3–6	9–93 × 2.5–4	2–4	PCA	Liu et al. (2025)
		26–45 × 10–17	3–6	4.5–65 × 2.5–4	2–4	V8A	
***A. hainanensis* sp. nov**.	**Obclavate to long-obclavate**	**24–53 × 10–18**	**3–6**	**7–33 × 3–5**	**4–7**	**PCA**	**This study**
		**32–55 × 12–20**	**4–7**	**22–40 × 3–5**	**4–7**	**V8A**	
*A. tomato*	Ellipsoid to long-ovoid	39–65 × 13–22	6–9	60–105 × 2	solitary	Host	Simmons (2007)
*A. nelumbicola*	–	24–37 × 9–16	–	10–43 × 2–3	up to 15 units	–	Zhang and Gao (2000)
	Narrow-ovoid, long-ovoid, or elongate-ellipsoid	30–50(–60) × 10–14	4–7	Absent or 10–40(–50) × 3–4	3–5	PCA	Guo et al. (2024)
	Elongate-ellipsoid to obclavate	33–49(–56) × 10–14	5–7	Absent or 10–45(–58) × 3–4	4–5	V8A	
*A. oryzicola*	Narrow-obclavate, obclavate, or elongate-ellipsoid	20–48 × 9–16	1–4	4–39 × 2.5–4	1–3	PCA	Liu et al. (2025)
		18–56 × 9–16	2–6	4–39 × 3–4	Solitary or 2–3	V8A	
*A. jacinthicola*	Short ellipsoid to ovoid, occasionally obclavate	9–28(–32) × 12–15	3–7	–	–	Host	Dagno et al. (2011)
***A. unguiculatae* sp. nov**.	**Ovate to narrowly obclavate**	**30–55 × 10–19**	**2–5**	**11–21 × 3–4**	**Solitary or 2–5**	**PCA**	**This study**
		**30–53 × 11–19**	**3–6**	**10–25 × 2.5–4.5**	**4–5**	**V8A**	

Notes: PCA, potato carrot agar; V8A, V8 juice agar. Boldface in the body of the table indicates the species described in this study and their corresponding morphological data. Missing data are indicated by an en dash (–).

#### Alternaria unguiculatae

Taxon classificationFungiPleosporalesPleosporaceae

S.X. Zhou & J.X. Deng sp. nov.

9C14367B-C705-507D-8140-AF1CC7F5F127

863710

Fig. 3

##### Etymology.

Named after the host plant Vigna unguiculata (cowpea), from which the pathogenic fungus was isolated.

**Figure 3.**
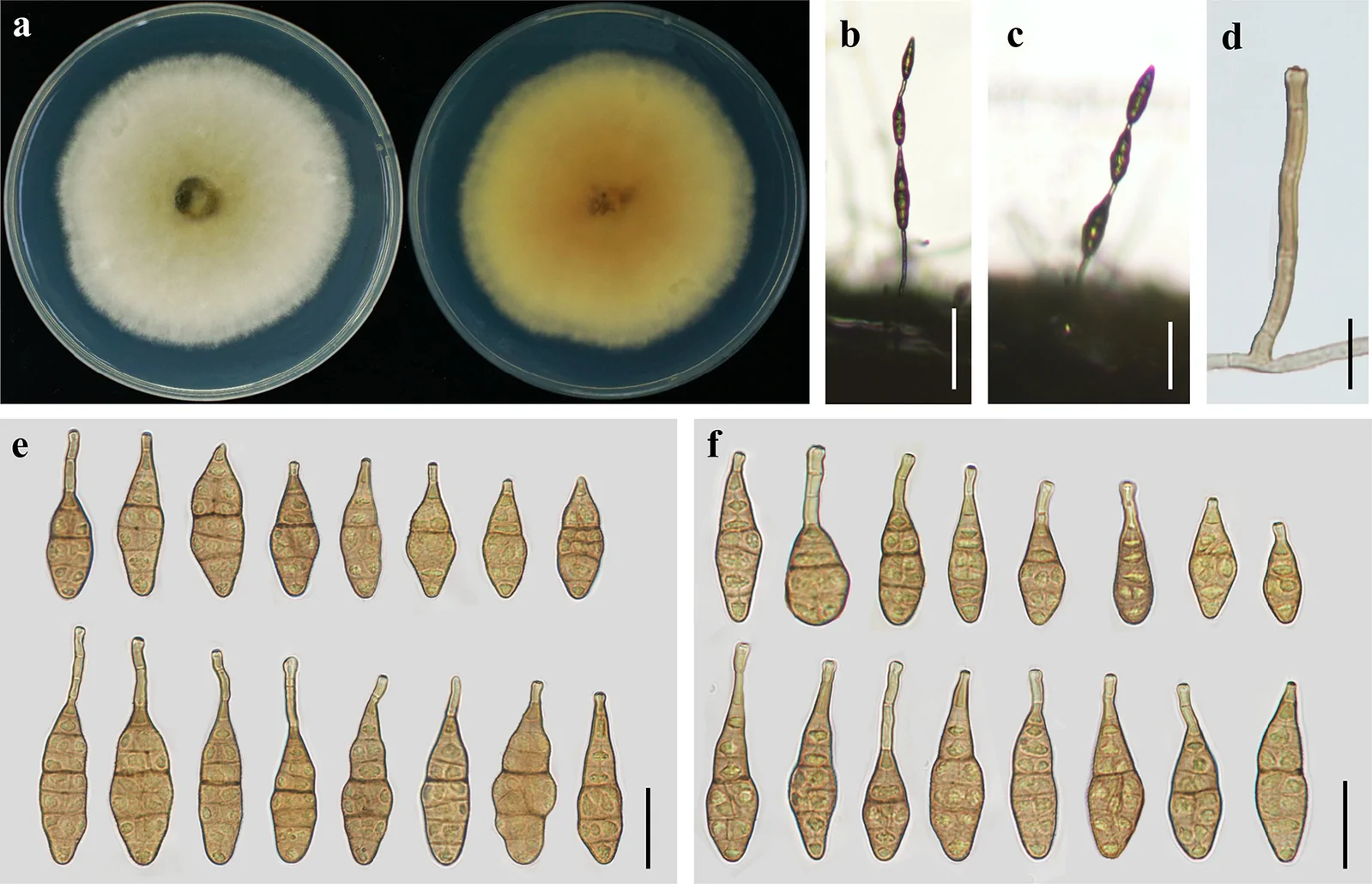
Morphology of *Alternaria unguiculatae* sp. nov. (YzU 241429). **a**. Colony on PDA for 7 days at 25 °C; **b, c**. Sporulation on PCA; **d**. Sporulation on V8A; **e**. Conidia on PCA; **f**. Conidia on V8A. Scale bars: 50 µm (**b, c, d**); 25 µm (**e, f**).

##### Type.

China • Hainan Province, Haikou City, from diseased leaves (cowpea); September 2024; S.X. Zhou; holotype YzU-H-2024-091A; ex-type culture YzU 241429; duplicate culture YzU 241428, derived from the same original isolate as the ex-type culture.

##### Description.

***Colonies*** on PDA (7 days at 25 °C in the dark) reached 48–52 mm in diameter, circular, floccose to cottony surface and abundant aerial mycelium, an irregularly serrate margin, Obverse grayish-white to sulphur yellow; reverse pure yellow to luteous. ***Conidiophores*** were brown, solitary, straight to slightly curved, arising from submerged or aerial hyphae, thin-walled, smooth, septate, and terminating in a single apical conidiogenous locus. ***Conidia*** on PCA solitary or in unbranched chains of 2–5, ovate to narrowly obclavate, 30–55 × 10–19 µm, yellow-brown, smooth, with 2–5 transverse and 0–1 longitudinal/oblique septa; median transverse septum often constricted and slightly thickened, basal cell rounded to subtruncate, apex tapered; some conidia bearing a cylindrical beak or pseudobeak continuous with the conidial body, occasionally slightly swollen at the tip, 11–21 × 3–4 µm. On V8A, sporulation is stable, conidial chains are longer (4–5 conidia) with morphology similar to those on PCA but slightly increased septation (3–6 septa); conidia measure 30–53 × 11–19 µm, beaks are cylindrical or pseudobeaked, slightly swollen apically, 10–25 × 2.5–4.5 µm.

##### Notes.

Phylogenetically, strain YzU 241429 formed a well-supported, independent lineage and was closest to *Alternaria jacinthicola* (MLBS = 98.1%, BYPP = 1.00). Comparative nucleotide sequence analyses showed that strain YzU 241429 differed from the included strains of *A. jacinthicola* at two positions in *Alt a 1* and one position in *RPB2* (Suppl. material 1: table SS2). No nucleotide differences were detected between the ex-type strain of *A. jacinthicola* in *GAPDH* or *EndoPG*. Morphologically, Dagno et al. (2011) reported conidia of *A. jacinthicola* measuring 9–28(–32) × 12–15 µm on PCA. In comparison, *A. unguiculatae* produces longer conidia, measuring 30–55 × 10–19 µm on the same medium, and beaks measuring 11–21 × 3–4 µm; quantitative beak dimensions were not reported for *A. jacinthicola* (Table 2).

## Discussion

Recent host-focused surveys have continued to reveal previously unrecognized diversity of small-spored taxa in *Alternaria* sect. *Alternaria* (Gou et al. 2022, 2023; Aung et al. 2024; Liu et al. 2025). Collectively, these studies indicate that species diversity within *Alternaria* sect. *Alternaria* remains incompletely resolved when sampling is focused on particular hosts, regions, or crop systems, even though the section has already been placed within a relatively stable phylogenetic framework (Woudenberg et al. 2015; Li et al. 2023). In this study, the recognition of *Alternaria unguiculatae* sp. nov. and *A. hainanensis* sp. nov. from cowpea extends this ongoing pattern in *Alternaria* sect. *Alternaria* further indicates that host-associated diversity in this section remains insufficiently documented.

At present, records of *Alternaria* associated with *Vigna unguiculata* and related genera remain limited. Previously reported taxa include *A. cassiae* from cowpea in South Africa (La Grange and Aveling 1998), *A. malorum* from cowpea leaf spot in Iran (Atashi Khalilabad and Fotouhifar 2022), *A. vignae* from *V. unguiculata* in China (Huang et al. 2022), and *A. alternata* from yardlong bean in India (Roy et al. 2024). The two species in this study provide genomic data for cowpea-associated *Alternaria*. In the multilocus phylogeny, *A. hainanensis* was recovered close to *A. zeae* and allied taxa, but it can be distinguished by a combination of conidial size, conidial chain length, septation, and beak development (Table 2). *Alternaria unguiculatae* was resolved close to *A. jacinthicola* but differs from that species by producing larger conidia and more distinctly developed beaks (Table 2). Across the comparator cultures included in the present analyses, *A. hainanensis* consistently differed from both cultures of *A. zeae* at three nucleotide positions in *GAPDH* and four positions in *RPB2*, whereas *A. unguiculatae* consistently differed from all three cultures of *A. jacinthicola* at two positions in *Alt a 1* and one position in *RPB2*. These substitutions are interpreted as lineage-associated character-state differences rather than as a numerical threshold sufficient on its own for species delimitation. In addition to the sequence differences detected at the loci examined, comparison with the seven phylogenetically related species included in Table 2 provides further support for the recognition of both taxa as distinct species, with each characterized by a distinctive combination of conidial dimensions, septation, chain formation, beak morphology, and colony pigmentation. Although yellowish pigmentation has also been reported in *A. jacinthicola* (Luo et al. 2018), this character alone is insufficient for species delimitation and should therefore be interpreted together with conidial morphology and phylogenetic evidence. These comparisons indicate that the recognition of the two new taxa is supported not by any single morphological character alone but by the congruence between multilocus phylogenetic placement and comparative morphology. In summary, the present study expands the known diversity of *Alternaria* associated with *Vigna unguiculata* and reinforces the value of combining detailed morphological comparison with multilocus phylogenetic evidence for delimiting closely related species in small-spored *Alternaria*.

## Supplementary Material

XML Treatment for Alternaria hainanensis

XML Treatment for Alternaria unguiculatae

## Additional information

### Artificial Intelligence (AI) use

The authors accept full responsibility for the content of the manuscript, including the disclosure of any use of AI.

No AI tools were used in the preparation of this manuscript.

### Author ORCIDs

S.-X. Zhou https://orcid.org/0009-0000-1581-6924

J.-H. Huang https://orcid.org/0009-0004-3164-5642

S.-Q. Chen https://orcid.org/0009-0003-7533-921X

Q. Deng https://orcid.org/0009-0000-4097-1520

P.-P. Yan https://orcid.org/0009-0007-5674-338X

J.-X. Deng https://orcid.org/0000-0001-7304-5603

### Data availability

All of the data that support the findings of this study are available in the main text or Supplementary Information.

## Supplementary materials

Supplementary material 1 Supplementary tables Data type docx Explanation note **table SS1**. Nucleotide differences and their base positions observed in the *GAPDH* and *RPB2* loci between *Alternaria zeae* and *Alternaria hainanensis*. **table SS2**. Nucleotide differences and their base positions observed in the *Alt a 1*, OPA10-2 and *RPB2* loci between *Alternaria jacinthicola* and *Alternaria unguiculatae*. https://binary.pensoft.net/file/1747727This dataset is made available under the Open Database License (http://opendatacommons.org/licenses/odbl/1.0/). The Open Database License (ODbL) is a license agreement intended to allow users to freely share, modify, and use this Dataset while maintaining this same freedom for others, provided that the original source and author(s) are credited. Shen-Xian Zhou, Jia-Hui Huang, Shi-Qiang Chen, Qian Deng, Cai-Hua Shi, Quan-Zhou Wen, Pian-Pian Yan, Jian-Xin Deng
