# Supplementary material for: Two novel species of Alternaria (Pleosporales, Pleosporaceae) from cowpea (Fabaceae) in China
Source: MycoKeys. 2026 Aug 18;138:329–44. doi: 10.3897/mycokeys.138.205866 (PMC13507769; doi:10.3897/mycokeys.138.205866)
Supplement: Supplementary file 1 [file mycokeys-138-329-s001.docx]

**Table S1.** Nucleotide differences and their base positions observed in the *GAPDH* and *RPB2* loci between *Alternaria zeae* and *Alternaria hainanensis*. The sequence of *A. zeae* YZU 231602^T^ was used as the reference for calculating base positions; alignment gaps were excluded. Nucleotides in parentheses represent intraspecific polymorphisms in *A. zeae* and were not treated as diagnostic differences. YzU 241394/241395 denotes duplicate culture accessions derived from one original isolate. No nucleotide differences were detected between *A. hainanensis* and *A. zeae* in *Alt a 1* or OPA10-2; no *EndoPG* sequence of *A. zeae* was available.

| Species | Culture | *GAPDH* | | | *RPB2* | | | |
| --- | --- | --- | --- | --- | --- | --- | --- | --- |
|  |  | 101^a^ | 266^a^ | 353^a^ | 54^a^ | 210^a^ | 228^a^ | 441^a^ |
| *Alternaria zeae* | YZU 231602^T^ | C | A | C | A | C | T | T |
| *Alternaria zeae* | YZU 231638 | (T) | (G) | C | A | C | T | T |
| *Alternaria hainanensis* | YzU 241394 | (T) | (G) | T | G | T | C | C |
| *Alternaria hainanensis* | YzU 241395 | (T) | (G) | T | G | T | C | C |

^a^Transition.

**Table S2.** Nucleotide differences and their base positions observed in the *Alt a 1*, OPA10-2 and *RPB2* loci between *Alternaria jacinthicola* and *Alternaria unguiculatae.* The sequence of *A. jacinthicola* CBS 133751^T^ was used as the reference for calculating base positions; alignment gaps were excluded. Nucleotides in parentheses represent polymorphic sites within *A. jacinthicola* and were not treated as diagnostic differences. No nucleotide differences were detected between *A. unguiculatae* and the ex-type culture of *A. jacinthicola* in *GAPDH* or *EndoPG*.

**A. *Alt a 1* and *RPB2***

| **Species** | **Culture** | ***Alt a 1*** | | | | | | ***RPB2*** |
| --- | --- | --- | --- | --- | --- | --- | --- | --- |
|  |  | 49^a^ | 145^a^ | 202^a^ | 295^a^ | 339^b^ | 363^a^ | 108^a^ |
| *Alternaria jacinthicola* | CBS 133751^T^ | **C** | **T** | **C** | **C** | **A** | **G** | **C** |
| *Alternaria jacinthicola* | CBS 878.95 | (T) | (C) | (T) | **C** | (T) | **G** | **C** |
| *Alternaria jacinthicola* | CPC 25267 | (T) | (C) | (T) | **C** | (T) | **G** | **C** |
| *Alternaria unguiculatae* | YzU 241428 | (T) | (C) | (T) | T | (T) | A | T |
| *Alternaria unguiculatae* | YzU 241429 | (T) | (C) | (T) | T | (T) | A | T |

**B. OPA10-2**

| **Species** | **Culture** | **OPA10-2** | | | | | | | | | | |
| --- | --- | --- | --- | --- | --- | --- | --- | --- | --- | --- | --- | --- |
|  |  | 1^a^ | 7^a^ | 45^a^ | 72^a^ | 79^b^ | 126^b^ | 159^a^ | 165^a^ | 195^a^ | 201^b^ | 216^a^ |
| *Alternaria jacinthicola* | CBS 133751^T^ | **T** | **C** | **A** | **T** | **A** | **G** | **C** | **T** | **A** | **T** | **T** |
| *Alternaria jacinthicola* | CBS 878.95 | **T** | **C** | **A** | **T** | **A** | **G** | **C** | **T** | **A** | **T** | **T** |
| *Alternaria jacinthicola* | CPC 25267 | **T** | **C** | **A** | (C) | **A** | **G** | **C** | **T** | **A** | **T** | **T** |
| *Alternaria unguiculatae* | YzU 241428 | C | T | G | (C) | T | T | T | C | G | G | C |
| *Alternaria unguiculatae* | YzU 241429 | C | T | G | (C) | T | T | T | C | G | G | C |

**B. OPA10-2 (continued)**

| **Species** | **Culture** | **OPA10-2** | | | | | | | | | |
| --- | --- | --- | --- | --- | --- | --- | --- | --- | --- | --- | --- |
|  |  | 222^a^ | 249^a^ | 261^a^ | 264^a^ | 294^a^ | 324^a^ | 447^a^ | 483^a^ | 513^a^ | 516^a^ |
| *Alternaria jacinthicola* | CBS 133751^T^ | **G** | **C** | **T** | **T** | **T** | **T** | **G** | **T** | **A** | **A** |
| *Alternaria jacinthicola* | CBS 878.95 | **G** | **C** | **T** | **T** | **T** | **T** | **G** | **T** | **A** | **A** |
| *Alternaria jacinthicola* | CPC 25267 | **G** | **C** | **T** | **T** | **T** | **T** | **G** | **T** | **A** | **A** |
| *Alternaria unguiculatae* | YzU 241428 | A | T | C | C | C | C | A | C | G | G |
| *Alternaria unguiculatae* | YzU 241429 | A | T | C | C | C | C | A | C | G | G |

^a^Transition;

^b^Transversion.
